# S18. PREDICTING PROGNOSIS IN PATIENTS WITH FIRST EPISODE PSYCHOSIS USING AUDITORY P300: A 1-YEAR FOLLOW-UP STUDY

**DOI:** 10.1093/schbul/sby018.805

**Published:** 2018-04-01

**Authors:** Kim Minah, Lho Kyung-jin, Lee Tak Hyung, Kwak Yoo Bin, Kwon Jun Soo

**Affiliations:** 1 Seoul National University College of Medicine; 2 Seoul National University College of Natural Science

## Abstract

**Background:**

Although early intervention is crucial for favorable outcome in patients with schizophrenia, development of biomarkers for predicting prognosis of psychotic disorder still requires more research. This study aims to investigate whether auditory P300 predict prognosis in patients with first episode psychosis (FEP) during 1-year of follow-up period.

**Methods:**

Twenty-four patients with FEP were examined with auditory P300 at baseline, and their clinical status were re-assessed after 1 year. Multiple regression analysis was used to identify factors predictive of prognosis in FEP patients during the follow-up period.

**Results:**

In the multiple regression analysis, P300 amplitude at CPz significantly predicted later improvement of Positive and Negative Syndrome Scale (PANSS) total, positive and general sub-scores. Improvement of Global Assessment of Functioning (GAF) and Brief Psychiatric Rating Scale (BPRS) scores were predicted by baseline P300 amplitude at CPz.

**Discussion:**

P300 may be a possible predictor of improvement in symptoms and functional status, as well as overall psychiatric status in patients with FEP. Future study with larger sample and longer follow-up period is needed to confirm the findings of the current study.

